# Adaptation of a LGBTQ Aging Dementia Education Intervention in Senior Living

**DOI:** 10.1093/geroni/igaf122.548

**Published:** 2025-12-31

**Authors:** Rajean Moone, Morgan Wright

**Affiliations:** University of Minnesota, Minneapolis, Minnesota, United States; University of Minnesota, Minneapolis, Minnesota, United States

## Abstract

Lesbian, gay, bisexual, transgender, and queer (LGBTQ) older adults have historically faced discrimination in long-term services and supports (LTSS), leading many to fear or avoid care or conceal their identities. At the same time, they often have a greater need for LTSS due to limited family or friend caregivers. In 2008, advocates, academics, state officials, and aging service providers launched the nonprofit Training to Serve (TTS) to improve LTSS providers’ understanding of LGBTQ aging. In 2024, the University of Minnesota began a randomized control trial to evaluate the effectiveness of the in-person TTS intervention, a new online version, and a control group in nursing homes and assisted living. This paper examines the community-engaged approach used to adapt the TTS curriculum for LTSS staff, including expanded content on Alzheimer’s and related dementias within LGBTQ communities. The curriculum development process integrated expertise from LGBTQ aging researchers and input from older LGBTQ community members to ensure cultural relevance. Additionally, the paper explores implementation strategies in 57 senior care facilities and presents findings from the evaluation study. Understanding how to adapt culturally relevant training is essential to improving care quality for LGBTQ residents in nursing homes and assisted living.

